# Prospective randomized trial of tumor-treating fields with chemoradiation in newly diagnosed glioblastoma

**DOI:** 10.1093/noajnl/vdag106

**Published:** 2026-04-24

**Authors:** Dror Limon, Felix Bokstein, Deborah T Blumenthal, Zvi Ram, Rachel Grossman

**Affiliations:** Radiation Oncology Unit, Davidoff Cancer Center, Rabin Medical Center, Petah-Tikva, Gray Faculty of Medical and Health Sciences, Tel Aviv University, Tel Aviv, Israel; Department of Oncology, Tel-Aviv Medical Center, Gray Faculty of Medical and Health Sciences, Tel Aviv University, Tel Aviv, Israel; Department of Oncology, Tel-Aviv Medical Center, Gray Faculty of Medical and Health Sciences, Tel Aviv University, Tel Aviv, Israel; Department of Neurosurgery, Tel-Aviv Medical Center, Gray Faculty of Medical and Health Sciences, Tel Aviv University, Tel Aviv, Israel; Department of Neurosurgery, Rambam Health Care Campus, Rappaport Faculty of Medicine, Technion, Haifa, Israel

**Keywords:** newly diagnosed glioblastoma, randomized clinical trial, survival, tumor treating fields

## Abstract

**Background:**

Tumor-treating fields (TTFields) have demonstrated efficacy in newly diagnosed glioblastoma (ndGBM) when combined with adjuvant temozolomide (TMZ). Preclinical studies suggest that  TTFields enhance the therapeutic effect of radiotherapy (RT), providing a rationale for concurrent administration with radiotherapy (RT). This phase 2 randomized trial evaluated the addition of TTFields to standard RT and TMZ, followed by adjuvant  TMZ with  TTFields, in patients with ndGBM.

**Methods:**

Patients were randomized prior to chemoradiation to receive RT and TMZ with or without concurrent TTFields, followed by maintenance  TMZ with  TTFields in both groups. The evaluable cohort, defined as an exploratory analysis, included patients who completed at least 4-weeks of RT (≥40Gy) and  TTFields in the experimental arm the primary endpoint, based on the intention-to-treat (ITT) population, was 1-year progression-free survival (PFS12). Secondary endpoints included PFS, overall survival (OS) and safety.

**Results:**

Sixty-six patients were randomized (experimental *n* = 32; control *n* = 34). In the ITT population, PFS12 favored the experimental group (28.7% vs 17.3%) but did not reach statistical significance (*P* = .146). Median PFS was 5.4 vs 4.13 months (*P* = .14) and median OS was 18.5 vs 16.5 months (*P* = .95) respectively. In the evaluable cohort (*n* = 54), PFS12 favored experimental group (37.5% vs 17.3%, *P* = .06), with a significantly longer median PFS (9.9 vs 4.1 months, *P* = .016). Median OS favored the experimental group (25.9 vs 16.6 months, *P* = .308). Treatment was well tolerated, with adverse events similar between groups.

**Conclusions:**

Early integration of  TTFields with chemoradiation in ndGBM is safe, feasible. Although the primary endpoint was not met, observed survival trends support further evaluation in larger multicenter trials to confirm the potential benefit of concurrent  TTFields with chemoradiation.

Key Points

- Concurrent  TTFields with chemoradiation is feasible, safe and improved efficacy in evaluable patients
- Although the primary endpoint, PFS12 was not met, survival trends favor  TTFields during chemoradiation, warranting validation in multicenter trials.

Importance of the Study

This phase 2 randomized trial provides the first prospective evidence supporting the early integration of Tumor Treating Fields (TTFields) with standard radiotherapy and temozolomide (RT/TMZ) in newly diagnosed IDH-wildtype glioblastoma. In the intention-to-treat analysis, the primary endpoint of 12-month progression-free survival (PFS12) numerically favored the concurrent  TTFields arm (28.7% vs 17.3%), although this difference did not reach statistical significance. These findings suggest that concurrent  TTFields with chemoradiation is both feasible and well tolerated, with a favorable safety profile. The trial provides preliminary clinical evidence supporting earlier TTFields initiation in the treatment sequence and sets the foundation for larger confirmatory studies to validate efficacy and define optimal implementation in routine neuro-oncologic practice.

Tumor treating fields (TTFields) is a noninvasive anticancer therapy that selectively target rapidly dividing cancer cells by delivering continuous low-intensity (∼1-3 V/cm), intermediate-frequency (100-500 kHz) alternating electric fields via noninvasive transducer arrays applied locoregionally on the skin.^1^ TTFields have been shown to prolong survival in patients with newly diagnosed glioblastoma (ndGBM), leading to its approval by the FDA for ndGBM after surgery and chemoradiotherapy, in addition to adjuvant temozolomide (TMZ).^2–4^

The FDA approval was based on positive results of the phase III EF-14 clinical trial that compared the addition of  TTFields to adjuvant  TMZ versus  TMZ alone in patients with ndGBM following surgery and chemoradiotherapy.^2^^,^^3^ The trial was terminated early after planned interim analysis demonstrated significant survival benefit. Patients receiving  TTFields and  TMZ achieved longer median progression-free survival (PFS) of 6.7 vs 4.0 months and median overall survival (OS) of 20.9 vs 16.0 months compared with TMZ alone.^2^^,^^3^ Long-term follow-up confirmed durable benefit, with 2-year survival rates of 43% versus 31%, and 5-year survival of 13% versus 5%.^2^^,^^3^ TTFields were generally well tolerated, with dermatological adverse events (AEs) being the most frequent treatment-related toxicity, while quality of life was not adversely affected.^4^

TTFields exert their cytotoxic effects through multiple pathways, including disruption of mitotic spindle assembly, interference with microtubules and septin fibers, inhibition of DNA damage repair, impairment of migration and invasion, and induction of autophagy.^5–14^ Preclinical studies in glioma models further demonstrated radiosensitizing effects, mediated by inhibition of double-stranded DNA repair via BRCA and Fanconi anemia (FA) pathways, resulting in mitotic catastrophe and reduced cell viability.^15^^,^^16^ These findings support a potential therapeutic synergy between  TTFields, radiotherapy (RT), and  TMZ. The safety of combining  TTFields with standard chemoradiotherapy was first evaluated in a phase I feasibility study of 10 patients with ndGBM,^17^ followed by the SPARE trial, which incorporated scalp-sparing RT with concurrent  TMZ and  TTFields.^18^ Both studies demonstrated feasibility and safety, with only mild dermatological toxicity of the scalp comparable to that reported in EF-14.

Based on these preclinical and early clinical findings, we initiated a randomized phase II trial to assess the efficacy and safety of TTFields administered concurrently with RT and TMZ in patients with ndGBM.

## Methods

### Study Population

Eligible patients were ≥18 years old with histologically confirmed supratentorial glioblastoma (WHO grade IV astrocytoma, IDH-wild type^19^) and a Karnofsky Performance Status (KPS) ≥70. All patients underwent maximal safe resection or biopsy and were scheduled to receive chemoradiation. IDH status was assessed by histology and immunohistochemistry. Molecular testing for rare IDH mutations was not performed routinely. Exclusion criteria included infratentorial tumors, early progression before initiation of RT/TMZ, IDH mutation, and severe comorbidities. The trial was approved by the institutional review board (IRB) and conducted in accordance with the Declaration of Helsinki (ClinicalTrials.gov identifier: NCT03869242). Written informed consent was obtained from all patients.

### Study Design and Randomization

**Figure 1.**
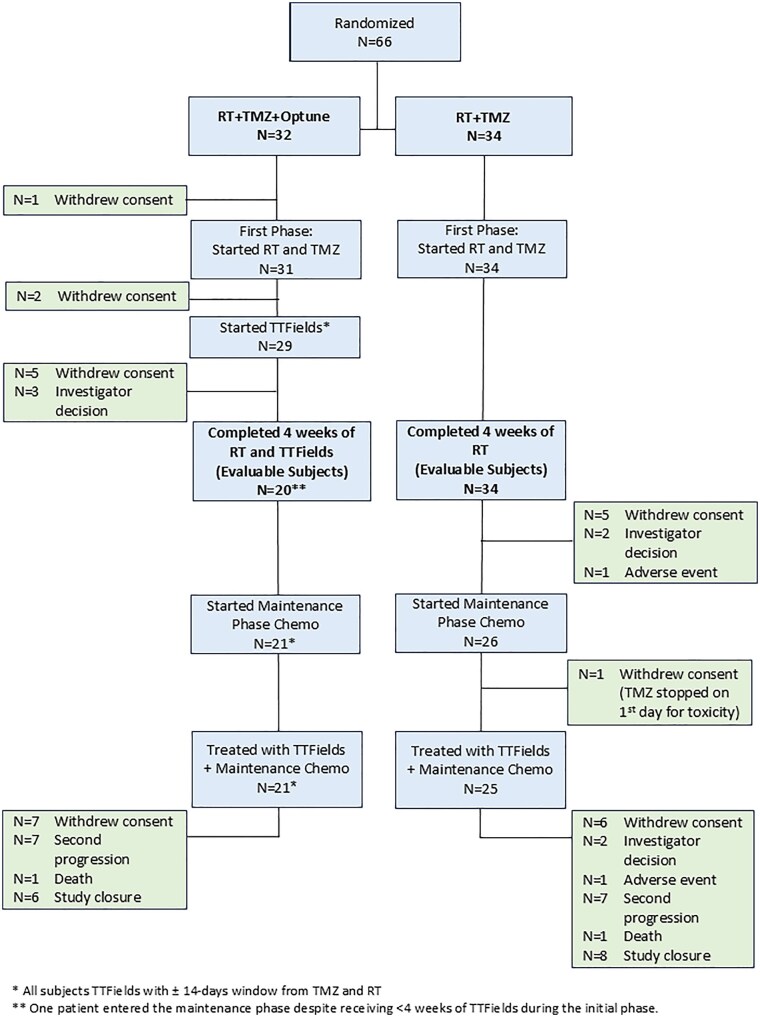
Consort flow diagram of patient enrollment.

This single-center, open-label, phase II randomized controlled trial was conducted between January 2019 and August 2021. Patients were randomized 1:1 to receive (1) standard RT and  TMZ (control group) or (2) RT and  TMZ plus TTFields (experimental group) for 6 weeks (initial phase) followed by maintenance TMZ with concurrent  TTFields for both groups (maintenance phase) (Figure 1). Evaluable patients were defined as those who completed at least 4 weeks of RT (≥40 Gy), and in the experimental arm, completion of TTFields during this period was additionally required. This criterion was applied to ensure assessment of the true effect of concurrent   TTFields and RT. Randomization was stratified by extent of resection (EOR); (biopsy, partial, or gross total), O6-methylguanine-DNA methyltransferase (MGMT) promoter methylation status (methylated, unmethylated, unknown), and age (≤65 vs >65 years), using variable block sizes. This open-label study did not mask treatment allocation, consistent with previous   TTFields clinical trials.^2–4^

RT consisted of local irradiation with 2 Gy per fraction to a total of 60 Gy, according to EORTC/NCIC protocols.^20^ Patients were instructed to remove the transducer arrays before every radiotherapy session. Concomitant TMZ was administered at 75 mg/m^2^/day during RT. Maintenance   TMZ was given at 150-200 mg/m^2^/day for 5 days every 28 days for 6 cycles, with extension permitted at the treating physician’s discretion in the absence of toxicity. Second-line therapy for progression (e.g. re-operation, re-irradiation, radiosurgery, and systemic therapy including bevacizumab) was administered at the physician’s discretion.

TTFields therapy could be continued until second radiological progression or clinical deterioration, for a maximum of 24 months.

### TTFields Procedures

TTFields were delivered using the NovoTTF-200A device (Novocure Inc., United States) via 2 transducer arrays (9 electrodes each) placed on the shaved scalp. Array layouts were determined using NovoTAL software, based on tumor location and head dimensions. Arrays were replaced twice weekly by patients or with support by device specialists. Patients were instructed to maintain ≥18 hours/day of TTFields therapy (≥75% compliance). Usage was recorded automatically by the device, uploaded biweekly, and reported to investigators as mean compliance.

### Follow-up and Assessments

Baseline evaluation was performed 2 weeks post-surgery. Follow-up visits occurred one month later and then every 2 months until the second progression. Assessments included MRI scans (per RANO criteria^21^) neurological evaluation, performance status, physical examination, hematology, and biochemistry. If clinical deterioration was suspected, an unscheduled MRI was obtained. After treatment discontinuation, patients were followed until death. Safety was assessed continuously, and AEs were graded according to CTCAE v5.0.

### Outcomes

The primary endpoint of this study was to evaluate whether the addition of TTFields to RT and TMZ improves PFS at 12 month (PFS12). The null hypothesis (H0) assumed no improvement with TTFields, while the alternative hypothesis (H1) tested for superiority of the experimental arm:


H0:PFS12(experimental)≤PFS12(control)

H1:PFS12(experimental)>PFS12(control)

where PFS12 (experimental) is the Kaplan-Meier estimate of the 12-month PFS rate in the experimental arm, and PFS12 (control) is the corresponding estimate in the control arm.

Secondary endpoints included OS, PFS, PFS at 6 months (PFS6), 1- and 2-year survival rates, radiological response, and AEs. Pre-specified subgroup analyses were performed for those who underwent gross total resection (GTR) and according to device compliance (>75% vs ≤75%). Exploratory post-hoc analyses were conducted for patients who were progression-free prior to maintenance therapy. OS was defined as time from diagnosis to death from any cause. PFS was defined as time from randomization to disease progression or death. Patients alive without progression were censored at last evaluable tumor assessment. Deaths occurring >3 months after last tumor assessment were censored at the date of assessment.

### Statistical Analysis

Categorical demographics and treatment characteristics were compared using Fisher’s exact test. Continuous variables were compared with the Wilcoxon Rank-Sum test due to non-normal distributions. PFS and OS were assessed using two-sided log-rank tests at an α level of 0.05, stratified by the experimental arm or within subgroups. Medians, confidence intervals (CIs), and rates were estimated using the Kaplan-Meier method, with comparisons made via a one-sided normal approximation test. Hazard ratios with 95% CIs were calculated using a stratified Cox proportional hazards model, with stratification variables introduced as covariates. Time-to-event analysis included censoring subjects without events. Some subgroups had prolonged median survival times with upper 95% confidence limits reported as not evaluable. Overall response rates’ 95% CI used the Clopper-Pearson method. Analyses covered patients treated with radiotherapy and TTFields alongside maintenance chemotherapy. PFS and OS in subgroups were post-hoc analyses. All analyses were conducted using SAS version 9.4 (SAS Institute, Cary, NC, United States).

## Results

### Demographics

Between January 2019 and August 2021, patients with newly diagnosed, histologically confirmed glioblastoma, IDH wild type were recruited from a tertiary medical center. A total of 69 patients were randomized to receive TTFields plus RT/TMZ (experimental group) or RT/TMZ alone (control group), followed by maintenance TMZ plus TTFields in both groups. Three patients were excluded due to disease progression prior to randomization, leaving 66 patients in the analysis cohort (experimental *n* = 32; control *n* = 34; Figure 1).

Eleven patients (34%) in the experimental group (8 withdrawal consent and 3 disease progression) discontinued before completing 4 weeks of RT and TTFields and before entering the maintenance phase. One patient entered the maintenance phase despite receiving <4 weeks of TTFields during the initial phase. In the control group, all 34 patients completed 4 weeks of RT. The Evaluable Population consisted of patients who completed 4 weeks of RT (and TTFields in the experimental arm), including 20 patients in the experimental group and 34 patients in the control group (Figure 1). Baseline characteristics were balanced across groups.

### Treatment Description

In the ITT experimental group, the median TTFields treatment duration was 9.8 months (interquartile range [IQR] 4-13) with median device usage of 76.2% (IQR: 67-86). In the experimental group of the evaluable cohort, the median TTFields treatment duration was 11.0 months (IQR: 9-15) with median device usage of 75.3% (IQR: 67-86). Among the 11 patients who were not considered evaluable in the experimental group, the median TTFields usage was 79.9%. During the initial treatment phase, the median TTFields duration was 2.6 (IQR: 2-3) and 2.7 months (IQR: 2-3) with median usage of 82.8% (IQR: 72-87) and 82.2% (IQR: 69-87) for the ITT and evaluable cohorts, respectively. In the maintenance phase, TTFields was used for a median of 8 months (IQR: 7-13), with median usage of 71.4% (46-85) and 72.1 (50-86) in the first 6 months for the ITT and evaluable cohorts, respectively (Table 1). In the control group, TTFields was applied for a median of 8.3 months (IQR: 4-18), with median usage of 76.3% (IQR: 68-89), during the first 6 months. A monthly average of ≥18 hours/day (≥75% daily use) was achieved by 42.9% of the experimental group in ITT and evaluable cohorts and 53.8% of the control group during the maintenance phase. Systemic therapy was switched to bevacizumab after progression in 8 patients (38%) from the experimental group and 13 (52%) from the control group (for both ITT and evaluable cohorts).

**Table 1.** Summary of demographic characteristics in the ITT cohort

Characteristic	RT+TMZ+ TTFields (*N* = 32)	RT+TMZ (*N* = 34)	*P* value
Age (Years), Mean (SD)	60.0 (11.78)	62.7 (8.85)	.290
Female, *n* (%)	10 (31.3%)	16 (47.1%)	.216
KPS, *n* (%)	90.0 (70-100)	90.0 (70-100)	.888
70-80	10 (31.3%)	10 (29.4%)	
90-100	18 (56.3%)	18 (52.9%)	
Time from diagnosis to randomization (days), Mean (SD)	34.5 (14.38)	31.9 (8.66)	.773
Extent of resection, *n* (%)			.932
Biopsy	4 (12.5%)	5 (14.7%)	
Partial resection	6 (18.8%)	5 (14.7%)	
Gross total resection	22 (68.8%)	24 (70.6%)	
MGMT status, *n* (%)			.440
Methylated	13 (40.6%)	10 (29.4%)	
Non methylated	19 (59.4%)	23 (67.6%)	
Unknown	0	1 (2.9%)	
Lesion type, *n* (%)			.122
Single	21 (65.6%)	29 (85.3%)	
Multifocal	10 (31.3%)	4 (11.8%)	
Duration of treatment with TMZ (Months), Median (IQR)	3.2 (1-8)	4.4 (1-7)	.979
Number of bevacizumab cycles, Median (IQR)	11.5 (11-15)	7.0 (3-13)	.102
Duration of treatment with TTFields (Months), Median (IQR)	9.8 (4-13)	8.3 (4-18)	.704
Average TTFields usage (%), Median (IQR)	76.2 (67-86)	82.4 (74-91)	.127
Duration of treatment with TTFields during first phase (Months), Median (IQR)	2.6 (2-3)	N/A	
Average TTFields usage during first phase (%), median (IQR)	82.8 (72-87)	N/A	
Duration of treatment with TTFields during maintenance phase (months), Median (IQR)	8 (7-13)	8.3 (4-18)	.991
Average usage during 6 months of maintenance phase, median (range), Median (IQR)	71.4 (46-85)	76.3 (68-89)	.290
Average usage group during first 6 months of maintenance phase, *n* (%)			.561
<75%	12 (57.1%)	12 (46.2%)	
≥75%	9 (42.9%)	14 (53.8%)	
Underwent re-operation, *n* (%)	8 (25%)	9 (26.5%)	1.000

**Table 2.** PFS rate for the ITT cohort

	Survival rate (%) (95% CI)			
Time point (Months)	RT + TMZ + TTFields	RT + TMZ	Absolute risk difference (95% CI)	*P* value
6	46.6 [28.2; 63.1]	39.9 [23.5; 55.9]	6.7 [−17.9; 31.3]	.297
9	43.0 [25.1; 59.8]	27.6 [13.8; 43.3]	15.4 [−8.2; 39.0]	.1
12	28.7 [13.8; 45.5]	17.3 [6.6; 32.2]	11.4 [−9.8; 32.7]	.146
18	28.7 [13.8; 45.5]	17.3 [6.6; 32.2]	11.4 [−9.8; 32.7]	.146
24	19.1 [6.7; 36.2]	3.5 [0.3; 14.9]	15.7 [−1.2; 32.5]	.034

In the ITT cohort, patients in the experimental group received TMZ for median of 3.2 months (IQR: 1-8) and 11.5 (IQR: 11-15) bi-monthly bevacizumab cycles, while those in the control group received TMZ for median of 4.4 months (IQR: 1-7) and 7 months (IQR: 3-13) bevacizumab cycles. In the evaluable cohort, patients in the experimental group received TMZ for a median of 6.7 months (IQR: 3-8) and 11.5 (IQR: 11-15) bi-monthly bevacizumab cycles (Table 1).

### Patient Disposition

All the 20 patients in the experimental group continued the maintenance phase. Eight (23.5%) in the control group (5 withdrawal consent, 3 disease progression) discontinued the study before the maintenance phase.

Thirty-two of the 54 evaluable patients (59%) discontinued the study, mainly due to second progression (25%) or withdrawal of consent (35%). Median study duration was 11 months for the experimental group and 8.7 months for the control group. Disease progression occurred in 16 patients (80%) in the experimental group and 32 (94%) in the control group. Three patients in the experimental group and 11 patients in the control group experienced progression during the initial phase. After progression, 30% of experimental and 26.4% of control patients underwent re-operation.

### PFS and OS in the ITT Cohort

**Figure 2.**
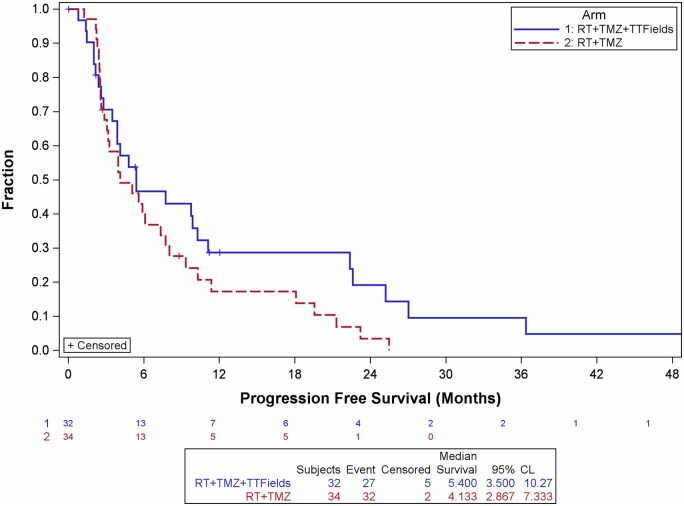
Kaplan-Meier plot showing progression-free survival among ITT patients. Hazard ratio = 0.668 [0.390; 1.143] log rank *P* = .137.

**Figure 3.**
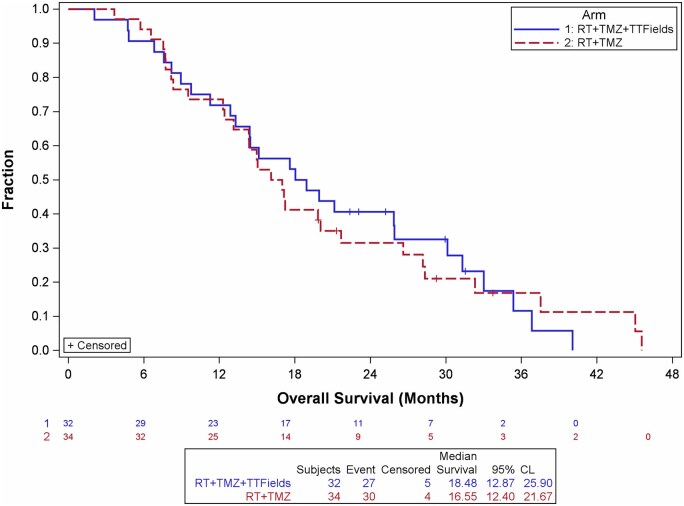
Kaplan-Meier plot of overall survival in ITT patients. Hazard ratio = 0.983. [0.578; 1.672] Log Rank *P* = .948.

In the ITT population, the predefined primary endpoint, PFS12, was higher in the experimental group compared with the control group (28.7% vs 17.3%, respectively), although this difference did not reach statistical significance (*P* = .15). Median PFS was 5.4 months (95% CI: 3.5-10.3) in the experimental group and 4.1 months (95% CI: 2.9-7.3) in the control group (HR = 0.67, 95% CI: 0.39-1.14; *P* = .14) (Table 2, Figure 2). Median OS was 18.5 months (95% CI: 12.9-25.9) in the experimental group versus 16.5 months (95% CI: 12.4-21.7) in the control group (HR = 0.98, 95% CI: 0.58-1.67; *P* = .95) (Figure 3). Two-year OS rates were 40.6% and 31.5% in the experimental and control groups, respectively (*P* = .22).

### PFS and OS in the Evaluable Cohort

In the evaluable cohort, PFS12 was higher in the experimental arm compared with the control arm (37.5% vs 17.3%), showing a favorable trend that did not reach statistical significance (*P* = .06). Median PFS was significantly longer in the experimental group (9.9 months, 95% CI: 4.1-22.6) than in the control group (4.1 months, 95% CI: 2.9-7.3; HR = 0.47, 95% CI: 0.25-0.88; log-rank *P* = .016; Supplementary Figure S1). Median OS was 25.9 months (95% CI: 15.1-31.3) in the experimental group versus 16.5 months (95% CI: 12.4-21.7) in the control group (HR = 0.73, 95% CI: 0.39-1. 34; *P* = .3; Supplementary Figure S2). Two-year OS rates were 55% and 31.5% in the experimental and control groups, respectively (*P* = .04), Supplementary Table S1.

### Subgroup Analyses

*TTFields usage ≥75%:* In high-adherence patients, median PFS was 11.1 vs 6.7 months (HR = 0.62, *P* = .32) and OS was 31.3 vs 18.5 months (HR = 0.48, *P* = .16) for experimental and control groups, respectively. Two-year OS was 78% vs 34% (*P* = .01; Supplementary Table S1).

*GTR subgroup*: Among patients who underwent GTR, median PFS was 9.9 vs 6.1 months (HR = 0.63; *P* = .27). Median OS was significantly longer in the experimental group (25.9 vs 15.0 months; HR = 0.35; *P* = .02), with 2-year OS rates of 54% vs 22% (*P* = .03).

*Biopsy or STR subgroup*: Among patients who underwent biopsy or STR, median PFS was 10.4 vs 4.6 months (HR = 0.32; *P* = .04). Median OS was significantly longer in the experimental group (25.9 vs 15.0 months; HR = 0.35; *P* = .02), with 2-year OS rates of 54% vs 22% (*P* = .03).

### Safety

All patients experienced at least 1 AE of any cause. Grade 3-5 AEs were reported in 28% of patients in the experimental group and 20.6% in the control group in the ITT cohort (Table 3, Supplementary Table S2), and were primarily attributed to chemotherapy, radiotherapy, or the underlying disease rather than to TTFields. The most frequent AEs overall were fatigue (54%), headache (50%), lymphopenia (43%), nausea (43%), and asthenia (41%).

**Table 3.** Summary of adverse events during RT (ITT)

Category severity	RT+TMZ+ TTFields (*N* = 32)	RT+TMZ (*N* = 34)	All patients (*N* = 66)
Subjects with AE during RT by maximum CTCAE grade	29 (90.6%)	31 (91%)	60 (90.9%)
Grade 1—Mild	15 (46.8%)	21 (62%)	36 (54.5%)
Grade 2—Moderate	12 (37.5%)	8 (23%)	20 (30.3%)
Grade 3—Severe	0	1 (3%)	1 (1.5%)
Grade 4—Life threatening	0	0	0
Grade 5—Fatal	0	0	0
Unknown	2 (6.3%)	1 (3%)	3 (4.5%)
Subjects with study device-related AE during RT by maximum CTCAE grade	11 (34.3%)	0	11 (16.6%)
Grade 1—Mild	7 (21.8%)	0	7 (10.6%)
Grade 2—Moderate	3 (9.4%)	0	3 (4.5%)
Grade 3—Severe	0	0	0
Grade 4—Life threatening	0	0	0
Grade 5—Fatal	0	0	0
Unknown	1 (3.1%)	0	1 (1.5%)
Subjects with chemotherapy-related AE during RT by maximum CTCAE grade	21 (65.6%)	25 (74%)	46 (69.6%)
Grade 1—Mild	16 (50%)	19 (56%)	35 (53%)
Grade 2—Moderate	5 (15.6%)	5 (15%)	10 (15.1%)
Grade 3—Severe	0	1 (3%)	1 (1.5%)
Grade 4—Life threatening	0	0	0
Grade 5—Fatal	0	0	0
Subjects with radiation therapy-related AE during RT by maximum CTCAE grade	18 (56.2%)	23 (68%)	41 (62.1%)
Grade 1—Mild	13 (40.6%)	22 (65%)	35 (53%)
Grade 2—Moderate	4 (12.5%)	1 (3%)	5 (7.6%)
Grade 3—Severe	0	0	0
Grade 4—Life threatening	0	0	0
Grade 5—Fatal	0	0	0
Unknown	1 (3.1%)	0	1 (1.5%)

Serious AEs (SAEs) occurred in 33% of patients in the experimental group and 36% in the control group. Only one SAE (folliculitis) was considered related to TTFields. Treatment discontinuation due to neurological deterioration occurred in one patient in each group.

Overall, TTFields-related AEs were reported in 12 patients (57%) in the experimental group and 15 patients (60%) in the control group, consisting mainly of mild-to-moderate skin reactions.

## Discussion

This phase 2 randomized clinical trial provides the first prospective evidence that adding TTFields to standard RT and TMZ, followed by adjuvant TMZ and TTFields, may improve outcomes in ndGBM.

In the ITT population, median PFS was 5.4 months in the experimental group versus 4.1 months in the control group (*P* = .14), with PFS12 rates of 28.7% and 17.3%, respectively (*P* = .14). Because the modest sample size led to a high rate of early censoring, which could mask treatment effects, we performed an evaluable cohort analysis. This included patients who completed at least 4 weeks of RT (≥40Gy) and TTFields, if randomized to the experimental arm.

Participant discontinuation was notable: 20 of 66 patients withdrew after randomization and an additional 13 during maintenance, mainly due to practical aspects of device use. This is consistent with prior TTFields trials, in which discontinuation rates of 23% in EF-11^22^ and 15% in EF-14^3^ were reported, largely due to non-compliance or handling difficulties. Concurrent administration with RT and TMZ in the present study may have contributed to the higher withdrawal rate. These findings emphasize that future randomized trials should account for high dropout rates when planning sample sizes to ensure the ITT population is adequately powered. While ITT analysis remains the gold standard for preserving the benefits of randomization, per-protocol analyses may provide exploratory insights into the device’s effect. In the evaluable cohort, median PFS was significantly longer in the experimental group (9.9 months) compared with the control group (4.1 months, *P* = .016), with a clinically meaningful hazard ratio of 0.47. The primary endpoint, PFS12, also favored the experimental arm (37.5% vs 17.3%), though the difference did not reach statistical significance (*P* = .06). OS demonstrated a consistent benefit for the experimental arm beginning approximately 7 months after randomization, with median OS of 25.9 versus 16.6 months. While this OS difference did not reach statistical significance, the magnitude of benefit is notable in the context of glioblastoma outcomes. Randomization was stratified by EOR,^23^ MGMT promoter methylation,^24^ and age, ensuring balanced baseline characteristics. Importantly, this trial randomized patients before initiation of chemoradiation, unlike the EF-14 trial where enrollment occurred only after completion of chemoradiation. This design captured patients with early tumor progression,^25^ yielding a more clinically representative cohort but also contributing to shorter PFS in the control arm compared with EF-14.

Although the proportion of MGMT-methylated versus unmethylated tumors appeared numerically imbalanced, statistical testing confirmed balance between groups. Consistently, Table 1 for the ITT population shows balanced MGMT status (*P* = .714), in line with MGMT being a stratification factor during randomization. Thus, the efficacy trends are unlikely to be explained by MGMT status or other baseline factors. Still, the modest sample size precludes definitive conclusions, underscoring the need for larger trials with sufficient power to permit multivariate analyses.

The results are biologically consistent with preclinical data showing that TTFields enhance the effect of RT by disrupting homologous recombination and impairing DNA damage repair, thereby increasing radiosensitivity. The earlier initiation of TTFields in the experimental group (median 2.7 months earlier than in controls) may also have contributed to the observed benefit. Treatment was feasible and well tolerated, with no increase in systemic toxicity compared with RT/TMZ alone. Dermatological AEs were limited to mild-to-moderate scalp reactions, in line with prior TTFields studies.^2–4^^,^^17^ The benefit observed in the subgroup of evaluable patients who completed the full 4-week device therapy concurrently with radiotherapy and chemotherapy suggests that adequate treatment exposure may be critical for achieving clinical benefit. The study was designed with 12-month PFS as the primary endpoint and was not powered to detect an OS benefit. Given that PFS events occur earlier and the number of events was sufficient, a statistically significant PFS benefit could be observed, whereas longer follow-up and more deaths would be required to demonstrate a significant OS effect. In this subgroup, a substantial improvement in PFS was seen, supporting the biological rationale for combining the device with standard chemoradiotherapy. To inform the design of a future randomized study, we performed sample size estimations based on the observed survival rates in the ITT population. Assuming a 12-month PFS of 17.3% in the control group and 28.7% in the treatment group, approximately 282 patients (141 per arm) would be required to detect this difference with 80% power at a two-sided α of 0.05. Similarly, assuming a 2-year OS rate of 31.5% in the control group, approximately 662 patients (331 per arm) would be required under the same assumptions. These estimates correspond to hazard ratios of approximately 0.71 for PFS and 0.78 for OS and may help guide the design of future prospective randomized trials.

All patients had IDH wild-type glioblastoma. IDH status was evaluated by immunohistochemistry for IDH1 R132H; rare non-R132H IDH1 or IDH2 mutations may not have been captured. Although enrollment began before the 2021 WHO classification, which redefined glioblastoma as IDH wild-type only, this ensured a homogeneous study population and strengthens the interpretation of findings in this single-center trial.

The results are biologically consistent with preclinical data showing that  TTFields enhance the effect of RT by disrupting homologous recombination and impairing DNA damage repair, thereby increasing radiosensitivity. The earlier initiation of  TTFields in the experimental group (median 2.7 months earlier than in controls) may also have contributed to the observed benefit. Treatment was feasible and well tolerated, with no increase in systemic toxicity compared with RT/TMZ alone. Dermatological AEs were limited to mild-to-moderate scalp reactions, in line with prior TTFields studies.^2–4^^,^^17^

This trial builds upon our earlier phase 1 pilot, which demonstrated the feasibility and safety of concurrent TTFields with chemoradiation and showed promising efficacy signals.^17^ Together, these results provide preliminary clinical evidence supporting concurrent TTFields with RT as a potentially beneficial strategy in glioblastoma.

Limitations include the small sample size, single-center design, absence of a sham control during RT, and lack of power for subgroup analyses. The evaluable cohort may be subject to selection bias. In the experimental arm, patients who complete at least 4 weeks of concurrent  TTFields and radiotherapy likely represent a subgroup with more favorable clinical status or treatment tolerance.  This may partially contribute to the observed differences in efficacy between the evaluable and ITT populations, independent of the treatment effect. Nevertheless, the consistent trends across PFS and OS, coupled with strong biological rationale, support further investigation. Larger multicenter studies, including the ongoing TRIDENT trial, are expected to validate and extend these findings.

## Conclusions

This phase 2 randomized controlled trial provides prospective evidence that adding TTFields to standard RT and TMZ, followed by adjuvant TMZ and TTFields, is feasible and safe in newly diagnosed IDH wild-type glioblastoma. Although the study did not meet its primary endpoint in the intention-to-treat analysis, 1-year PFS numerically favored the experimental arm (28.7% vs 17.3%) without reaching statistical significance. The consistent efficacy trends together with the favorable safety profile support further evaluation in adequately powered randomized trials.

## Supplementary Material

vdag106_Supplementary_Data

## Data Availability

The data that support the findings of this study are available from the corresponding author upon reasonable request. Individual participant data are de-identified to protect patient privacy in accordance with institutional and ethical guidelines. Requests for data sharing will be reviewed by the study investigators and the institutional ethics committee to ensure compliance with data protection regulations.
