# Supplementary material for: Prospective randomized trial of tumor-treating fields with chemoradiation in newly diagnosed glioblastoma
Source: Neurooncol Adv. 2026 Apr 24;8(1):vdag106. doi: 10.1093/noajnl/vdag106 (PMC13228130; doi:10.1093/noajnl/vdag106)
Supplement: vdag106_Supplementary_Data [file vdag106_supplementary_data.zip › Supplementary Table and Figure Legends.docx]

**Supplementary Figure 1**: Kaplan–Meier Plot Showing Progression-Free Survival Among evaluable Patients. Hazard Ratio = 0.471 [0.253 ; 0.880] Log Rank P = 0.016

**Supplementary Figure 2**: Kaplan–Meier Plot of Overall Survival in evaluable Patients. Hazard Ratio = 0.726 [0.392 ; 1.347] Log Rank P = 0.308
