# Supplementary material for: Prospective randomized trial of tumor-treating fields with chemoradiation in newly diagnosed glioblastoma
Source: Neurooncol Adv. 2026 Apr 24;8(1):vdag106. doi: 10.1093/noajnl/vdag106 (PMC13228130; doi:10.1093/noajnl/vdag106)
Supplement: vdag106_Supplementary_Data [file vdag106_supplementary_data.zip › Supplementary Table 1_rev2 clear version.docx]

### **Supplementary Table 1**: OS and PFS rates summary for the evaluable cohort

| Endpoint (Rate (%) [95% CI]) | 12 Months | 18 Months | 24 Months |
| --- | --- | --- | --- |
| PFS  Experimental group (N=20)  Control group (N=34)  *Difference (Experimental-control), P-Value* | 37.5 [16.9; 58.2]  17.3 [6.6 32.2]  20.2 [-5.3; 45.8] 0.06 | 37.5 [16.9; 58.2] 17.3 [6.6; 32.2] 20.2 [-5.3; 45.8]  0.06 | 22.5 [6.3; 44.8]  3.5 [0.3; 14.9]  19.0 [-2.7; 40.8] 0.043 |
| OS  Experimental group (N=20)  Control group (N=34)  *Difference (Experimental-control), P-Value* | 90.0 [65.6; 97.4] 73.5 [55.3; 85.3] *16.5 [-3.3; 36.3] 0.052* | 75.0 [50.0; 88.7] 41.2 [24.8; 56.9] *33.8 [8.6; 59.0] 0.004* | 55.0 [31.3; 73.5] 31.5 [16.8; 47.5] *23.5 [-3.5; 50.4] 0.044* |
| PFS among pts with ≥75% usage  Experimental group (N=9)  Control group (N=14)  *Difference (Experimental-control), P-Value* | 44.4 [13.6 ; 71.9]  26.8 [7.3 ; 51.5]  *17.7 [-22.8 ; 58.1] 0.196* | 44.4 [13.6 ; 71.9]  26.8 [7.3 ; 51.5]  *17.7 [-22.8 ; 58.1] 0.196* | 22.2 [1.4 ; 58.8]  8.9 [0.6 ; 32.3]  *13.3 [-25.2 ; 51.8]*  *0.249* |
| OS among pts with ≥75% usage  Experimental group (N=9)  Control group (N=14)  *Difference (Experimental-control), P-Value* | 88.9 [43.3 ; 98.4]  92.9 [59.1 ; 99.0]  *-4.0 [-28.5 ; 20.6]*  *0.376* | 88.9 [43.3 ; 98.4]  50.0 [22.9 ; 72.2]  *38.9 [5.6 ; 72.2]*  *0.011* | 77.8 [36.5 ; 93.9]  34.3 [11.6 ; 58.7]  *43.5 [6.2 ; 80.8]*  *0.011* |
| PFS = progression free survival; OS = overall survival; PD = progressive disease | | | |
