# Supplementary material for: Prospective randomized trial of tumor-treating fields with chemoradiation in newly diagnosed glioblastoma
Source: Neurooncol Adv. 2026 Apr 24;8(1):vdag106. doi: 10.1093/noajnl/vdag106 (PMC13228130; doi:10.1093/noajnl/vdag106)
Supplement: vdag106_Supplementary_Data [file vdag106_supplementary_data.zip › Supplementary Table 2_rev.docx]

**Supplementary Table 2**: Summary of AEs by treatment arm (ITT)

| **Category Severity** | **RT+TMZ+** **TTFields (N=32)** | **RT+TMZ (N=34)** | **All Patients (N=66)** |
| --- | --- | --- | --- |
| **Subjects with AE by Maximum CTCAE Grade** | 30 (93.7) | 32 (94.1) | 62 (93.9) |
| Grade 1 - Mild | 6 (18.8) | 5 (14.7) | 11 (16.7) |
| Grade 2 - Moderate | 13 (40.6) | 20 (58.8) | 33 (50.0) |
| Grade 3 - Severe | 9 (28.0) | 6 (17.7) | 15 (22.7) |
| Grade 4 - Life Threatening | 0 | 0 | 0 |
| Grade 5 - Fatal | 0 | 1 (2.9) | 1 (1.5) |
| Unknown | 2 (6.3) | 0 | 2 (3.0) |
