# Supplementary figures and images for: Prospective randomized trial of tumor-treating fields with chemoradiation in newly diagnosed glioblastoma
Source: Neurooncol Adv. 2026 Apr 24;8(1):vdag106. doi: 10.1093/noajnl/vdag106 (PMC13228130; doi:10.1093/noajnl/vdag106)

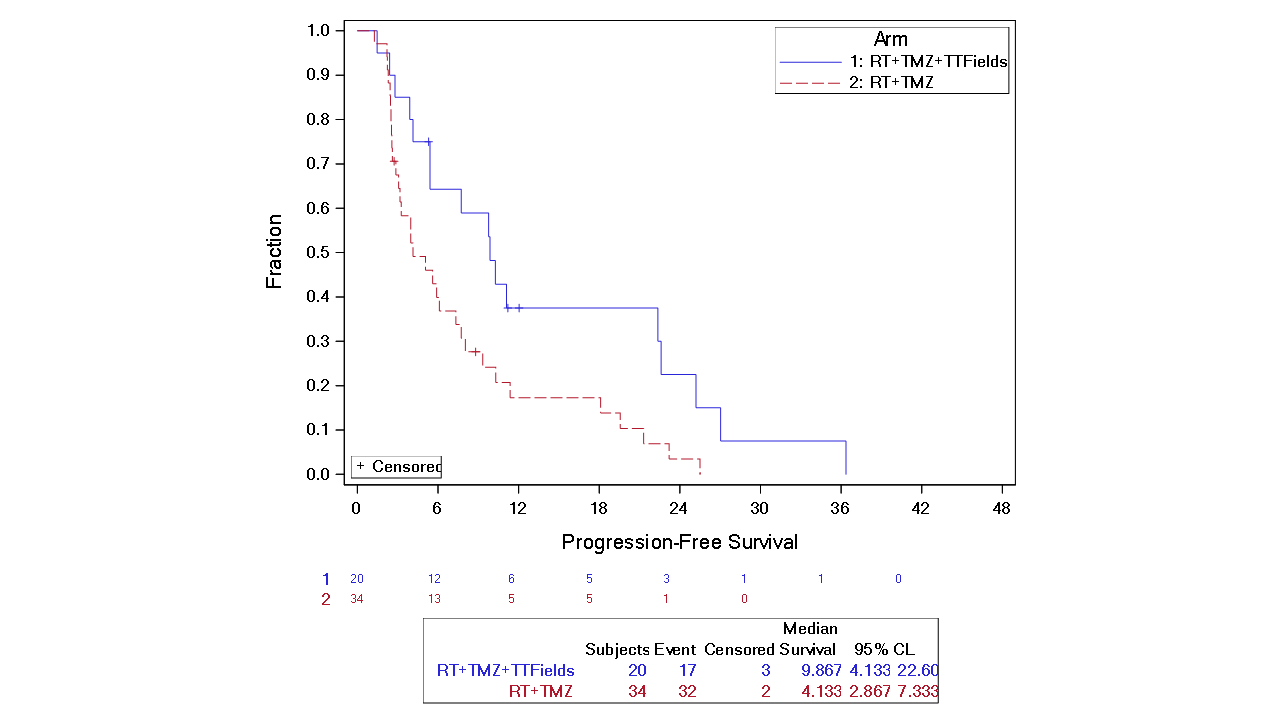

Supplement: vdag106_Supplementary_Data [file vdag106_supplementary_data.zip › Supplementary Figure 1 v1.tif]

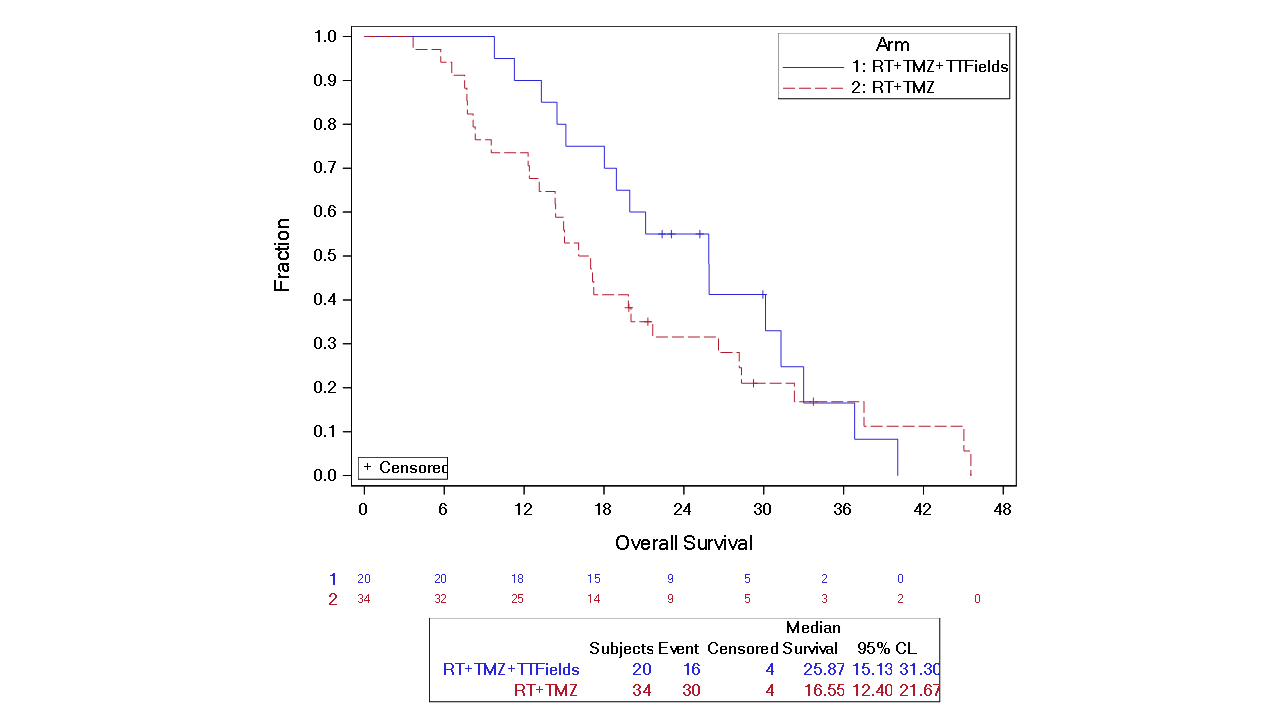

Supplement: vdag106_Supplementary_Data [file vdag106_supplementary_data.zip › Supplementary Figure 2 v1.tif]
